# Hyperthermic Intraperitoneal Chemotherapy in Gastric Cancer: Distinguishing Prophylactic and Therapeutic Roles Within Contemporary Multimodal Management

**DOI:** 10.7759/cureus.115175

**Published:** 2026-08-25

**Authors:** Saqer Dayis, Mohammed Alshaikh, Roaa Alosaimi, Saud Almutairi, Ebaa Jamjoom, Turki Alotaibi, Marwan Al-Hajeili, Shouki Bazarbashi, Mohamed Aseafan

**Affiliations:** 1 Section of Medical Oncology, Department of Internal Medicine, Security Forces Hospital Program, General Directorate of Medical Services, Ministry of Interior, Riyadh, SAU; 2 Oncology Clinical Pharmacy, Pharmaceutical Services, Security Forces Hospital Program, Ministry of Interior, Riyadh, SAU; 3 Section of Medical Oncology, Oncology Center, King Saud University Medical City, King Saud University, Riyadh, SAU; 4 Department of Medicine, King Abdulaziz University, Jeddah, SAU; 5 Oncology Center, King Faisal Specialist Hospital and Research Centre, Riyadh, SAU

**Keywords:** cytoreductive surgery, gastric cancer, hyperthermic intraperitoneal chemotherapy, peritoneal metastasis, peritoneal recurrence, prophylactic hipec

## Abstract

Peritoneal metastasis (PM) remains one of the most clinically important determinants of poor outcome in gastric cancer (GC), given the limited efficacy of systemic therapy within the peritoneal compartment. This has driven sustained interest in hyperthermic intraperitoneal chemotherapy (HIPEC), administered after cytoreductive surgery (CRS), as a strategy to improve locoregional disease control, although its role remains controversial in both prophylactic and therapeutic settings.

A narrative review of the literature was performed using published randomized controlled trials, prospective cohort studies, retrospective comparative analyses, systematic reviews, and meta-analyses evaluating HIPEC in GC. Particular emphasis was placed on biological rationale, patient selection, disease burden, completeness of cytoreduction, treatment protocols, perioperative morbidity, and oncologic outcomes. The prophylactic and therapeutic applications of HIPEC were evaluated separately to avoid conflating two biologically and clinically distinct questions.

In high-risk resectable disease, prophylactic HIPEC demonstrates a recurrent signal toward reduced peritoneal recurrence and improved disease-free survival, although supporting evidence remains heterogeneous and largely nonrandomized. In established PM, earlier studies and selected retrospective analyses suggested a survival signal with CRS-HIPEC; however, contemporary randomized trials, particularly GASTRIPEC-I and PERISCOPE II, have not demonstrated a convincing overall survival benefit and have highlighted important concerns regarding treatment-related morbidity and clinical trade-offs. Across both settings, outcomes appear highly dependent on patient selection, especially peritoneal cancer index, tumor biology, and the feasibility of complete cytoreduction.

Overall, HIPEC remains biologically compelling but clinically unconfirmed as routine care in GC. At present, prophylactic HIPEC should be regarded as investigational but promising, whereas therapeutic CRS-HIPEC for established gastric PM should remain restricted to highly selected patients managed in experienced centers and, ideally, within clinical trials or prospective registries.

## Introduction and background

Gastric cancer (GC) remains one of the leading causes of cancer-related mortality worldwide despite advances in systemic therapy, perioperative management, and multidisciplinary care. According to GLOBOCAN 2020 estimates, GC is the fifth most commonly diagnosed malignancy and the fourth leading cause of cancer-related death globally. Its burden is not evenly distributed, with particularly high incidence rates in East Asia, while outcomes in metastatic disease remain poor across regions [1].

Among the patterns of disease progression in GC, peritoneal metastasis (PM) is one of the most clinically consequential and prognostically adverse manifestations. Peritoneal dissemination may be present at initial diagnosis or may develop after apparently curative-intent surgery, and in both scenarios it is associated with a marked deterioration in survival. Population-based and institutional series consistently show that the peritoneum is not only a frequent site of metastasis at presentation but also a dominant site of recurrence following surgery for locally advanced disease, especially in tumors characterized by serosal invasion, diffuse histology, or positive peritoneal cytology [2-4].

The poor outcome associated with PM is partly explained by the biologic and pharmacologic isolation of the peritoneal compartment. Although systemic therapy remains the foundation of treatment in advanced disease, drug penetration into the peritoneal cavity is limited by the peritoneal-plasma barrier. This pharmacokinetic limitation has prompted interest in locoregional strategies capable of delivering higher effective drug concentrations directly to peritoneal surfaces. Hyperthermic intraperitoneal chemotherapy (HIPEC), administered immediately after cytoreductive surgery (CRS), represents one such strategy and combines maximal macroscopic tumor reduction with regionally intensified chemotherapy under hyperthermic conditions [5,6].

Yet the key clinical question remains unsettled: does this biologically rational approach translate into meaningful benefit in modern practice? Importantly, the HIPEC literature in GC spans two fundamentally different clinical scenarios. The first is prophylactic HIPEC, used in patients without overt macroscopic PM but with clinicopathologic features that confer a high risk of future peritoneal recurrence. The second is therapeutic CRS-HIPEC, applied in patients with established peritoneal disease in an effort to improve survival through aggressive locoregional treatment. These two indications differ in disease burden, therapeutic intent, biologic plausibility, and expectations of benefit, and they should not be interpreted as interchangeable.

This distinction has become even more important in the contemporary era. Earlier studies and retrospective series generated enthusiasm for CRS-HIPEC in selected patients with gastric PM, but more recent randomized evidence has narrowed the scope for overly optimistic interpretation. In particular, GASTRIPEC-I and PERISCOPE II have forced a more critical reassessment of therapeutic HIPEC, while the prophylactic question remains open and biologically attractive [7,8].

The aim of this review is therefore not simply to summarize the literature on HIPEC in GC, but to evaluate its role within contemporary multimodal management by treating prophylactic and therapeutic applications as distinct clinical questions. In doing so, this review focuses on biological rationale, patient selection, technical considerations, morbidity, and the strengths and limitations of the key available studies, with particular attention to how recent evidence should influence present-day interpretation and future research priorities.

## Review

Methods: literature search strategy

A narrative review of the literature was conducted using published randomized controlled trials, prospective non-randomized studies, retrospective comparative analyses, systematic reviews, and meta-analyses evaluating HIPEC in GC. Particular emphasis was placed on studies addressing prophylactic HIPEC in high-risk resectable disease and therapeutic CRS-HIPEC in patients with established PM.

The review was structured to address four core questions: first, the biological and pharmacologic rationale for HIPEC in GC; second, the extent to which patient selection determines outcomes; third, whether prophylactic HIPEC provides clinically meaningful reductions in peritoneal recurrence and disease relapse; and fourth, whether therapeutic CRS-HIPEC confers an overall survival advantage in the era of modern systemic therapy.

Preference was given to higher-level evidence when available, particularly randomized trials and well-conducted comparative analyses. Because the prophylactic and therapeutic settings represent different clinical problems, evidence was synthesized separately for each. Narrative interpretation prioritized trial design, oncologic endpoints, disease burden, completeness of cytoreduction, and real-world applicability.

Current standard of care and the rationale for continued HIPEC investigation

Management of GC is fundamentally stage-dependent and requires coordinated multidisciplinary care integrating surgery, systemic therapy, and, in selected cases, radiotherapy. For patients with resectable disease, perioperative chemotherapy combined with gastrectomy and adequate lymphadenectomy represents the standard approach in most Western treatment paradigms, historically supported by MAGIC and later refined by FLOT4, which established fluorouracil, leucovorin, oxaliplatin, and docetaxel (FLOT) as the preferred perioperative cytotoxic backbone for fit patients with resectable disease. More recently, the MATTERHORN trial showed that adding perioperative durvalumab to FLOT improved event-free survival, with later reporting also demonstrating an overall survival advantage, further reshaping the perioperative landscape in resectable GC and gastroesophageal junction adenocarcinoma [4,9-13].

In the metastatic setting, systemic therapy remains the cornerstone of treatment, but the first-line landscape is increasingly biomarker-driven. In human epidermal growth factor receptor 2 (HER2)-positive advanced disease, the ToGA trial established trastuzumab plus chemotherapy as first-line standard. This approach was subsequently strengthened by KEYNOTE-811, which demonstrated improved efficacy with the addition of pembrolizumab to trastuzumab and chemotherapy. More recently, HERIZON-GEA-01 has emerged as an important first-line HER2-positive study and may further alter the therapeutic landscape. These developments are highly relevant because they define the systemic-treatment context into which any locoregional strategy, including HIPEC, must now be integrated [11,14-16].

Within this broader framework, PM represents a distinct and particularly challenging clinical scenario. The efficacy of systemic therapy in the peritoneal compartment is constrained by pharmacologic limitations, as drug penetration across the peritoneal-plasma barrier is suboptimal. Consequently, even in patients with otherwise controlled systemic disease, peritoneal progression remains a frequent mechanism of treatment failure.

Current clinical guidelines, including the latest National Comprehensive Cancer Network (NCCN) guidance, do not recommend HIPEC as part of routine standard care for GC with PM. Instead, they emphasize that peritoneal-directed strategies such as CRS and HIPEC may be considered only in highly selected patients after multidisciplinary evaluation and preferably within experienced, high-volume centers. This cautious position reflects both the heterogeneity of the available evidence and the recognition that any potential benefit is likely restricted to a narrowly defined subgroup of patients [11].

The continued interest in HIPEC therefore arises not from established guideline endorsement, but from a persistent clinical gap. PM remains one of the most lethal patterns of disease progression in GC, and current systemic therapies do not adequately address this compartment. While CRS can remove macroscopic disease, it cannot reliably eliminate microscopic tumor deposits distributed across the peritoneal surface. HIPEC is intended to address this limitation by delivering high concentrations of cytotoxic agents directly to the peritoneal cavity in the immediate postoperative setting.

The challenge, however, lies in reconciling biological plausibility with clinical evidence. While the theoretical rationale for HIPEC is compelling, its real-world value must be assessed within the context of modern systemic therapy, evolving surgical standards, and the potential for treatment-related morbidity. This tension between promise and proof explains why HIPEC remains controversial and why its role continues to be actively investigated.

Biological rationale, pharmacologic basis, and procedural constraints

The rationale for HIPEC is grounded in the unique pharmacokinetic and biologic characteristics of intraperitoneal drug delivery. By administering chemotherapy directly into the peritoneal cavity, HIPEC achieves substantially higher local drug concentrations than would be feasible with systemic administration while limiting systemic exposure. This creates a favorable peritoneal-to-plasma concentration gradient and allows intensified regional therapy within a compartment that is otherwise difficult to treat effectively [5,6].

Agents commonly used in HIPEC, including cisplatin, mitomycin C, and oxaliplatin, possess pharmacologic properties that support intraperitoneal administration, such as relatively high molecular weight and slower systemic absorption. These features allow prolonged exposure of peritoneal surfaces to cytotoxic concentrations during the perfusion period [5,6].

Hyperthermia is a critical component of the HIPEC strategy. Elevating intraperitoneal temperatures to approximately 41-43°C enhances the cytotoxic effects of chemotherapy through multiple mechanisms, including increased cellular membrane permeability, improved intracellular drug uptake, inhibition of DNA repair pathways, and direct protein denaturation. Hyperthermia may also preferentially impair tumor cells, which often exhibit a less effective heat-shock response than normal tissues [5,6].

The timing of HIPEC administration is also biologically relevant. Delivery immediately after CRS targets residual microscopic disease at a point when tumor burden has been minimized and before viable tumor cells can reimplant and proliferate. This concept is particularly important in the prophylactic setting, where the primary objective is eradication of free or microscopic tumor cells rather than treatment of established macroscopic disease [5,6].

Despite these theoretical advantages, several important limitations must be acknowledged. The depth of tissue penetration achieved by intraperitoneal chemotherapy is limited, generally extending only a few millimeters from the peritoneal surface. As a result, HIPEC is ineffective against bulky residual disease. This limitation underscores the central importance of achieving complete or near-complete cytoreduction before HIPEC. In this context, HIPEC functions as an adjunct to surgery rather than a compensatory modality, and its efficacy is fundamentally dependent on surgical quality [5,6,17].

Another major challenge is the lack of standardization across HIPEC protocols. Substantial variability exists in drug selection, dosing strategies, perfusion temperature, duration, and technical approach, including open, closed, or semi-open techniques. For example, oxaliplatin-based regimens are typically administered over shorter durations with higher drug concentrations, whereas mitomycin C protocols often involve longer perfusion times. Cisplatin-based regimens may require specific hydration strategies to mitigate nephrotoxicity [6].

This heterogeneity complicates interpretation of the literature. Studies frequently refer to “HIPEC” as a unified intervention, although the underlying procedures may differ substantially in pharmacologic exposure and technical execution. Consequently, the existing evidence base represents a spectrum of related approaches rather than a single standardized treatment, which contributes significantly to ongoing uncertainty regarding clinical efficacy [6].

In addition to pharmacologic variability, procedural factors influence outcomes. CRS-HIPEC is a complex intervention requiring specialized surgical expertise and meticulous perioperative management. Operative duration, extent of resection, and institutional experience all affect both morbidity and oncologic outcomes. These considerations further reinforce that HIPEC is not a standalone therapy but part of a broader, highly specialized treatment strategy [6,17].

Patient selection: the central determinant of outcome

Across the HIPEC literature in GC, the most consistent finding is that outcomes are driven primarily by patient selection rather than by the HIPEC procedure itself. Differences in survival across studies are largely explained by variation in disease burden, tumor biology, and the ability to achieve complete cytoreduction. As such, appropriate patient selection is the critical determinant of whether HIPEC provides benefit or harm [6,17,18].

The peritoneal cancer index (PCI) is the most widely used quantitative measure of peritoneal disease burden. By assessing tumor distribution and lesion size across defined abdominal regions, PCI provides both a numerical estimate of disease extent and a surrogate marker of tumor dissemination. Numerous studies have demonstrated a strong inverse relationship between PCI and survival outcomes, and although reported cutoffs vary somewhat by cohort, they converge on a similar range. In the Italian multicenter series, patients with a PCI ≤6 had a median overall survival of 44.3 months versus 13.4 months for those with a PCI >6 [17]. The CYTO-CHIP propensity-matched analysis similarly found that long-term survival was rare beyond a PCI of 13, with the CRS-HIPEC cohort achieving benefit at a mean PCI of only 7.2 [18]. Other retrospective series and registries have reported comparable thresholds, with median survival of roughly 18-26 months for PCI 0-6, falling to 12-19 months for PCI 7-15, and 5-13 months for PCI values above 15-20 [6,17,18]. Taken together, most available data converge on a PCI in the range of 6-7 as the threshold below which patients derive the greatest benefit from CRS-HIPEC, with a PCI above approximately 12-13 generally considered a relative or absolute contraindication; values between these thresholds (PCI 7-12) represent an intermediate zone in which candidacy should be individualized based on cytoreduction feasibility and tumor biology. These figures are derived largely from non-randomized, single- or multi-institutional cohorts, and a universally validated cutoff has not been established; nonetheless, they provide clinicians with actionable numerical benchmarks rather than a qualitative instruction to "select carefully".

Equally important is the completeness of cytoreduction (CC). The CC score reflects the amount of residual disease following surgery and is one of the strongest predictors of long-term survival. Patients achieving CC-0 or CC-1 demonstrate markedly improved outcomes compared with those left with macroscopic tumor deposits. This reinforces the principle that HIPEC cannot compensate for inadequate surgery. Patients in whom complete cytoreduction is not achievable should not be considered appropriate candidates for CRS-HIPEC, as the addition of HIPEC does not overcome the adverse effect of residual disease [5,6,17].

Accurate preoperative staging is therefore essential. Conventional imaging modalities such as computed tomography and magnetic resonance imaging have limited sensitivity for detecting small-volume peritoneal disease and often underestimate disease extent. Diagnostic laparoscopy, frequently combined with peritoneal cytology, provides direct visualization of peritoneal surfaces and allows more accurate assessment of PCI. Failure to incorporate staging laparoscopy may lead to inappropriate patient selection, either by overestimating resectability or by missing diffuse disease that would preclude effective cytoreduction [4,6,11].

Tumor biology further influences outcomes. Diffuse-type histology, signet ring features, and poorly differentiated tumors are associated with more aggressive behavior and a higher propensity for peritoneal dissemination. These factors may limit the effectiveness of locoregional strategies even when technically feasible. Response to systemic therapy can also serve as a dynamic marker of tumor biology. Patients with stable or responsive disease during induction therapy are more likely to benefit from aggressive locoregional approaches, whereas those with rapidly progressive disease are unlikely to derive meaningful benefit.

In the prophylactic setting, the concept of selection shifts from disease extent to risk stratification. Patients without macroscopic peritoneal disease but with high-risk features, such as serosal invasion, positive cytology, diffuse histology, or extensive nodal involvement, are at increased risk of peritoneal recurrence and therefore represent potential candidates for preventive strategies such as HIPEC [11,19].

However, even within this high-risk group, careful consideration of patient fitness and perioperative risk remains essential. The potential benefit of prophylactic HIPEC must be balanced against the added morbidity of the procedure, particularly in the absence of definitive randomized evidence demonstrating an overall survival advantage.

Taken together, these considerations highlight that HIPEC is not a broadly applicable intervention but a highly selective strategy. Differences in PCI, resectability, tumor biology, and clinical context fundamentally alter the risk-benefit balance, and this variability likely explains much of the heterogeneity observed across published studies.

Prophylactic HIPEC in gastric cancer

The application of HIPEC in the prophylactic setting is based on the premise that eradication of microscopic peritoneal disease may be more effective than attempting to control established macroscopic metastasis. Patients with serosal invasion, positive peritoneal cytology, diffuse histology, or extensive nodal involvement are at particularly high risk of peritoneal recurrence, providing a biologic rationale for early locoregional intervention [19].

Among prospective data, the randomized study by Beeharry et al. demonstrated one of the clearest signals supporting this approach. In patients undergoing D2 gastrectomy for locally advanced GC, the addition of HIPEC significantly reduced peritoneal recurrence and improved disease-free survival without a statistically significant increase in major postoperative complications. These findings suggest that prophylactic HIPEC can be integrated safely in selected patients while altering recurrence patterns [20].

Subsequent studies have reproduced this pattern, although with less consistency in survival endpoints. Zhong et al. evaluated lobaplatin-based HIPEC in patients with advanced GC and reported a significant improvement in disease-free survival, accompanied by reduced peritoneal recurrence, but without a corresponding overall survival benefit. A later analysis focusing on T4 disease similarly demonstrated improved long-term disease-free survival, again without a statistically significant difference in overall survival [21,22].

Retrospective comparative data further support the concept that earlier intervention may be more effective than treatment in the metastatic setting. In the study by Rosa et al., prophylactic HIPEC was associated with the most favorable outcomes among three groups, prophylactic HIPEC, therapeutic HIPEC, and surgery alone, with improved long-term survival and lower peritoneal recurrence rates. However, the nonrandomized design introduces substantial selection bias, and these results should be interpreted cautiously [23].

At a broader level, systematic reviews and meta-analyses provide a more consistent signal, albeit with important limitations. The review by Brenkman et al., which included 11 comparative studies (>1,000 patients), demonstrated reduced peritoneal recurrence and improved disease-free survival, with a trend toward improved overall survival [19]. A more recent meta-analysis restricted to randomized trials has confirmed reductions in peritoneal relapse and improved disease control, although overall survival benefit remains variable [24]. Notably, these two analyses are not fully independent, as both draw substantially on the same core set of prophylactic HIPEC trials, so their apparent agreement reflects overlapping source data rather than two truly independent confirmatory bodies of evidence.

Additional local real-world data from Saudi Arabia provide useful regional context for interpretation of prophylactic HIPEC. In a retrospective cohort of patients with localized gastric or gastroesophageal junction cancer treated at a tertiary center, no statistically significant survival benefit was demonstrated for prophylactic HIPEC and intraoperative radiotherapy, and patients managed without prophylactic HIPEC/IORT showed numerically longer progression-free and overall survival. Although this analysis was not designed as a dedicated HIPEC efficacy study, it offers important local real-world evidence that does not confirm a clear benefit for prophylactic HIPEC/IORT in localized disease and further reinforces the importance of careful patient selection and prospective validation [25].

The most important ongoing effort to resolve these uncertainties is the GASTRICHIP trial, a randomized phase III study evaluating D2 gastrectomy with or without oxaliplatin-based HIPEC in high-risk patients [26]. By standardizing both patient selection and treatment protocol, this trial is expected to provide the highest-quality evidence regarding whether the observed improvements in disease-free survival translate into a clinically meaningful survival advantage. However, GASTRICHIP was designed and initiated in 2012-2013, predating FLOT and perioperative durvalumab as standard systemic backbones, and enrollment closed only after an extended accrual period, with mature five-year survival results still pending more than a decade after activation [9,12,13,26]. This delay is itself informative: it suggests that a trial protocolized under an older systemic-therapy paradigm risks the same obsolescence this review highlights elsewhere, since any disease-free survival benefit detected may reflect a treatment landscape that no longer represents current practice. GASTRICHIP should therefore be interpreted as an important but time-lagged data point rather than a fully contemporary test of HIPEC's role alongside modern perioperative regimens.

Key prophylactic HIPEC studies are summarized in Table 1.

**Table 1:** Key Prophylactic HIPEC Studies in Gastric Cancer HIPEC: Hyperthermic intraperitoneal chemotherapy

Study	Design	Population	HIPEC Regimen/Context	Key Outcomes	Limitations
Beeharry et al. [20]	Randomized study	Locally advanced gastric cancer	Cisplatin-based HIPEC after D2 gastrectomy	Reduced peritoneal recurrence and improved disease-free survival without a significant increase in major complications	Small, single-center study
Zhong et al. (2020) [21]	Cohort study	Advanced gastric cancer	Lobaplatin-based HIPEC	Improved disease-free survival and reduced recurrence; no clear overall survival benefit	Non-randomized design
Zhong et al. (2023) [22]	Retrospective study	T4 gastric cancer	Lobaplatin-based HIPEC	Improved 5-year disease-free survival; no significant overall survival difference	Retrospective design and selection bias
Rosa et al. [23]	Retrospective comparative study	Advanced gastric cancer	Prophylactic HIPEC vs therapeutic HIPEC vs surgery alone	Prophylactic HIPEC associated with the most favorable long-term outcomes and lower peritoneal recurrence	Significant risk of selection bias
Brenkman et al. [19]	Systematic review	High-risk gastric cancer	Mixed regimens	Consistent reduction in peritoneal recurrence and improved disease-free survival across studies; overall survival trend variable	Heterogeneity across included studies
Stefano et al. [24]	Meta-analysis	Mixed prophylactic and therapeutic gastric cancer populations	Mixed regimens	Pooled signal toward reduced peritoneal relapse and improved disease control	Protocol and population heterogeneity
GASTRICHIP [26]	Ongoing phase III trial	High-risk resectable gastric cancer	Oxaliplatin-based HIPEC	Results pending	Not yet reported

Taken together, the prophylactic HIPEC literature demonstrates a reproducible pattern: consistent reduction in peritoneal recurrence and frequent improvement in disease-free survival, but without definitive confirmation of overall survival benefit. The strength of the signal, combined with a biologically favorable setting of minimal residual disease, supports continued investigation. However, given the predominance of nonrandomized data and heterogeneity in treatment protocols, prophylactic HIPEC should currently be regarded as a promising but investigational strategy rather than standard practice.

Therapeutic CRS-HIPEC for established peritoneal metastasis

The application of CRS combined with hyperthermic intraperitoneal chemotherapy in patients with established PM from GC has been investigated for many years, but its interpretation has changed substantially with the emergence of contemporary randomized data. Earlier studies and retrospective analyses suggested that aggressive locoregional treatment might improve outcomes in selected patients. However, more recent evidence has challenged that view and has led to a more cautious, selective, and biologically constrained understanding of therapeutic CRS-HIPEC [7,27].

The randomized phase III trial by Yang et al. remains one of the foundational studies in this field. In that trial, patients with GC and peritoneal carcinomatosis were randomized to undergo CRS alone or CRS combined with HIPEC. Median overall survival was significantly longer in the CRS-HIPEC arm than in the surgery-alone arm, providing early proof of principle that intensified locoregional therapy could influence outcomes in this setting [27].

This signal was later reinforced by observational data, most notably the CYTO-CHIP study, a multicenter propensity score-matched analysis. In that study, CRS-HIPEC was associated with improved median overall survival and recurrence-free survival compared with CRS alone. Although the size and design of CYTO-CHIP make it one of the strongest real-world analyses in this area, it remains observational, and residual confounding cannot be excluded. Variables such as disease burden, surgical selection, response to prior therapy, and tumor biology are difficult to control fully in nonrandomized datasets and may have contributed to the observed benefit [18].

Contemporary randomized evidence has provided a more restrained interpretation. The GASTRIPEC-I trial evaluated the addition of HIPEC to CRS in patients with synchronous PM who had received systemic therapy. In contrast to earlier studies, no overall survival difference was observed between the two treatment arms, although progression-free survival improved in the HIPEC group. These results suggest that improved locoregional disease control does not necessarily translate into an overall survival advantage in a disease where systemic progression remains a dominant determinant of outcome [7].

The PERISCOPE II trial further sharpened this reassessment. In that study, systemic therapy alone was compared with gastrectomy plus CRS-HIPEC in patients with limited peritoneal disease or positive cytology following induction treatment. No overall survival advantage was demonstrated for the surgical-HIPEC strategy, while the CRS-HIPEC arm experienced higher rates of severe adverse events and treatment-related mortality. These findings are particularly important because they reflect a modern treatment context and directly confront the assumption that more aggressive locoregional treatment necessarily improves outcomes [8].

Meta-analyses continue to report pooled survival advantages associated with CRS-HIPEC, but these findings must be interpreted with caution. Many pooled analyses combine retrospective studies with earlier trials conducted in different systemic-therapy eras and under highly variable selection criteria. As a result, summary estimates may overstate benefit when compared with contemporary randomized evidence. The discrepancy between pooled positive signals and negative modern phase III trials remains one of the central interpretive challenges in this literature [24].

Key therapeutic CRS-HIPEC studies are summarized in Table 2.

**Table 2:** Key Therapeutic CRS-HIPEC Studies in Gastric Peritoneal Metastasis CRS: Cytoreductive surgery; HIPEC: hyperthermic intraperitoneal chemotherapy

Study	Design	Population	Comparator	Key Outcomes	Interpretation
Yang et al. [27]	Phase III randomized trial	Gastric cancer with peritoneal carcinomatosis	CRS alone vs CRS-HIPEC	Median overall survival improved with CRS-HIPEC	Foundational positive trial, but performed in a pre-modern systemic therapy era
CYTO-CHIP [18]	Propensity score-matched analysis	Limited peritoneal metastatic gastric cancer	CRS alone vs CRS-HIPEC	Improved overall survival and recurrence-free survival with CRS-HIPEC	Strong observational signal, but subject to selection bias
GASTRIPEC-I [7]	Phase III randomized trial	Synchronous peritoneal metastasis after systemic therapy	CRS alone vs CRS-HIPEC	No overall survival benefit; progression-free survival improved	Important contemporary negative overall survival trial
PERISCOPE II [8]	Randomized trial	Limited peritoneal disease / positive cytology	Systemic therapy alone vs gastrectomy plus CRS-HIPEC	No overall survival benefit; higher severe adverse events and treatment-related mortality in the CRS-HIPEC arm	Strong contemporary signal against routine therapeutic use
Contemporary meta-analyses [24]	Pooled analyses	Mixed gastric PM populations	Various	Pooled survival benefit signal	Interpretation limited by inclusion of retrospective and older studies

Taken together, the current evidence suggests that while CRS-HIPEC may improve disease control in highly selected patients, a reproducible overall survival benefit has not been demonstrated in the modern era. The therapeutic setting is characterized by higher tumor burden, more aggressive disease biology, and greater interaction with systemic progression, all of which limit the capacity of a locoregional intervention to alter the overall course of disease. For this reason, therapeutic CRS-HIPEC should not be considered standard treatment for GC with established PM. Rather, it should remain restricted to highly selected patients with favorable biology, limited disease burden, and a realistic likelihood of complete cytoreduction, ideally within experienced centers and in the context of clinical trials or prospective registries.

Morbidity, real-world applicability, and the cost of being aggressive

CRS combined with HIPEC represents one of the most extensive interventions in abdominal oncology, and its potential oncologic benefit must be interpreted in the context of substantial treatment-related morbidity. Across published series, major postoperative complications occur at meaningful rates, with variability largely driven by disease burden, extent of cytoreduction, institutional experience, and perioperative management [6,18]. The spectrum of complications includes anastomotic leakage, intra-abdominal sepsis, hemorrhage, renal toxicity-particularly with cisplatin-based regimens-and hematologic suppression related to intraperitoneal chemotherapy exposure [5,6].

The most contemporary randomized perspective on this issue comes from PERISCOPE II, in which the addition of CRS-HIPEC to systemic therapy was associated with a significantly higher rate of severe adverse events and treatment-related mortality without a corresponding overall survival advantage. These findings emphasize a critical clinical reality: improved locoregional disease control does not automatically translate into a net patient benefit when weighed against perioperative risk and the persistent threat of systemic progression [8].

An additional concern relates to the impact of postoperative recovery on the delivery of systemic therapy. Delays in initiating or resuming chemotherapy following CRS-HIPEC are common in patients experiencing complications or prolonged recovery. Given that systemic progression remains a dominant driver of mortality in C with PM, any interruption or delay in systemic therapy may offset gains achieved through locoregional control. This interaction between surgical morbidity and systemic disease dynamics is central to understanding why improved progression-free survival in trials such as GASTRIPEC-I did not translate into improved overall survival [4,7,11].

This concern is also reflected in local real-world data from Saudi Arabia. In a retrospective cohort of patients with localized gastric or gastroesophageal junction cancer treated at a tertiary center, nearly half received prophylactic HIPEC combined with intraoperative radiotherapy, yet no statistically significant survival benefit was observed, and patients managed without prophylactic HIPEC/IORT demonstrated numerically longer progression-free and overall survival. Although this was not a dedicated HIPEC efficacy study and should not be overinterpreted, it provides important local contextual evidence that the addition of aggressive locoregional modalities does not inherently guarantee better outcomes and reinforces the need for careful patient selection and real-world feasibility assessment [25].

Real-world applicability further complicates interpretation. The majority of favorable outcomes reported in the literature originate from high-volume, specialized centers with dedicated expertise in peritoneal surface malignancies. These institutions employ standardized surgical techniques, perioperative protocols, and multidisciplinary decision-making pathways that are not universally reproducible. As a result, outcomes achieved in these settings may not be generalizable to broader clinical practice. This has led major guidelines, including the NCCN, to recommend that CRS-HIPEC, if considered at all, should be restricted to carefully selected patients managed in experienced centers following multidisciplinary evaluation [11].

Ultimately, CRS-HIPEC represents a high-risk, high-complexity intervention in which the therapeutic margin is narrow. In the absence of a demonstrated overall survival benefit in modern randomized trials, the threshold for patient selection must remain stringent. The decision to proceed with CRS-HIPEC should therefore reflect a deliberate balance between potential oncologic benefit and the real cost of treatment-related morbidity.

Why the prophylactic question may be stronger than the therapeutic one

A consistent pattern across the HIPEC literature in GC is that the prophylactic application of HIPEC appears biologically more plausible and, in terms of disease control, more consistent than its use in established PM. This distinction must be stated plainly: to date, no study of any design, randomized or non-randomized, has demonstrated that prophylactic HIPEC prolongs overall survival; every supporting study has shown, at most, reduced peritoneal recurrence and improved disease-free survival, without a corresponding survival benefit. This is not primarily a matter of the strength of the evidence, but rather a distinction in what has actually been demonstrated, and readers should not equate a favorable disease-control signal with proof of a survival advantage. Fundamental differences in disease burden, timing of intervention, and the mechanism through which HIPEC exerts its effect help explain why the prophylactic setting has produced a more coherent disease-control signal, but they do not substitute for the missing survival data.

In the prophylactic setting, HIPEC is delivered in the absence of macroscopic peritoneal disease, targeting free intraperitoneal tumor cells and microscopic residual disease at the time of gastrectomy. Given that intraperitoneal chemotherapy penetrates tissue to a depth of only a few millimeters, this context is inherently more favorable for therapeutic efficacy (5,6]. The consistent reduction in peritoneal recurrence and improvement in disease-free survival observed across prophylactic studies supports this biological rationale [19-23].

In contrast, therapeutic HIPEC is applied after the development of established PM, where disease burden is higher, tumor distribution is more diffuse, and tumor biology is often more aggressive. Even with optimal cytoreduction, residual microscopic disease may be extensive, and systemic dissemination frequently coexists. Under these conditions, the capacity of HIPEC to meaningfully alter the natural history of disease is limited, as reflected in contemporary randomized trials that have failed to demonstrate an overall survival advantage despite improvements in progression-free survival [7,8].

Timing also matters. Prophylactic HIPEC is delivered at an early stage, before resistant tumor clones emerge and before peritoneal implants become established. Therapeutic HIPEC, by contrast, is typically applied after exposure to systemic therapy and often in a setting of treatment-resistant disease. This temporal difference further contributes to the observed disparity in outcomes.

Taken together, these biologic and clinical distinctions suggest that the prophylactic setting represents the more rational context for HIPEC investigation. This does not justify routine adoption in current practice, but it does explain why the prophylactic literature shows a more coherent and reproducible disease-control signal than the therapeutic literature. For that reason, ongoing and future trials focused on prophylactic HIPEC may ultimately prove more informative than continued broad extrapolation from the therapeutic setting.

Future directions

Future development of HIPEC in GC requires resolution of several key limitations that have historically constrained its clinical adoption. Foremost among these is the need to clearly separate prophylactic and therapeutic indications in both trial design and data interpretation. Combining these fundamentally different clinical scenarios has contributed significantly to heterogeneity and confusion within the literature.

Standardization of HIPEC protocols is equally critical. Existing studies demonstrate substantial variation in chemotherapeutic agents, dosing, temperature, duration, and delivery technique, limiting comparability and reproducibility. Establishing uniform, evidence-based protocols will be essential for generating interpretable data and defining best practice [6].

Integration with modern systemic therapy represents another major priority. Much of the HIPEC literature predates contemporary perioperative regimens and the incorporation of immunotherapy and targeted therapy. Future trials must reflect current standards, including biomarker-driven approaches and evolving perioperative immunotherapy strategies, to ensure relevance to modern clinical practice. In particular, the MATTERHORN results illustrate how rapidly the resectable-disease standard can change, and future HIPEC studies that fail to incorporate contemporary perioperative regimens risk becoming outdated before their findings can be translated into practice [4,11-13].

Advances in patient selection are also required. Beyond traditional clinical parameters such as PCI and completeness of cytoreduction, emerging molecular and biologic markers may improve identification of patients most likely to benefit from locoregional strategies. Dynamic assessment of response to systemic therapy may further refine selection criteria.

Finally, the results of ongoing randomized trials, particularly GASTRICHIP, are expected to provide critical evidence regarding the role of prophylactic HIPEC. These studies will determine whether improvements in disease-free survival translate into meaningful long-term outcomes and whether HIPEC can be integrated into standard multimodal treatment strategies [26].

A limitation of the current HIPEC literature is that much of the available evidence remains heterogeneous in design, heavily influenced by retrospective series, and difficult to compare because of nonuniform protocols, variable disease burden, and inconsistent systemic-therapy backbones. This literature-level limitation should temper interpretation of pooled survival signals and underscores the importance of contemporary, protocol-standardized randomized trials.

## Conclusions

The role of HIPEC in GC is best understood as two distinct clinical questions rather than a single therapeutic concept. In the prophylactic setting, there is a consistent and biologically plausible signal for reduced peritoneal recurrence and improved disease-free survival, although definitive phase III validation is still lacking. In the therapeutic setting, early encouraging results have been tempered by contemporary randomized evidence demonstrating no overall survival benefit and highlighting significant treatment-related morbidity. This caution is also supported by local real-world experience, which did not confirm a benefit for prophylactic HIPEC/IORT and further emphasizes that aggressive locoregional approaches should not be adopted outside careful selection and appropriate multidisciplinary context.

Accordingly, prophylactic HIPEC may be considered a promising but investigational strategy, while therapeutic CRS-HIPEC for established peritoneal metastasis should remain highly selective, centralized to experienced centers, and preferably conducted within clinical trials or prospective registries.
